# Recent Progress in Diboronic-Acid-Based Glucose Sensors

**DOI:** 10.3390/bios13060618

**Published:** 2023-06-04

**Authors:** Ke Nan, Yu-Na Jiang, Meng Li, Bing Wang

**Affiliations:** 1 Ningbo Key Laboratory of Biomedical Imaging Probe Materials and Technology, Ningbo Institute of Materials Technology and Engineering, Chinese Academy of Sciences, Ningbo 315201, China; 2 School of Pharmaceutical Sciences, Cixi Biomedical Research Institute, Wenzhou Medical University, Wenzhou 325035, China; 3 International Cooperation Base of Biomedical Materials Technology and Application, Ningbo Cixi Institute of Biomedical Engineering, Ningbo 315300, China

**Keywords:** diboronic acid, glucose sensors, recognition mechanism

## Abstract

Non-enzymatic sensors with the capability of long-term stability and low cost are promising in glucose monitoring applications. Boronic acid (BA) derivatives offer a reversible and covalent binding mechanism for glucose recognition, which enables continuous glucose monitoring and responsive insulin release. To improve selectivity to glucose, a diboronic acid (DBA) structure design has been explored and has become a hot research topic for real-time glucose sensing in recent decades. This paper reviews the glucose recognition mechanism of boronic acids and discusses different glucose sensing strategies based on DBA-derivatives-based sensors reported in the past 10 years. The tunable p*K_a_*, electron-withdrawing properties, and modifiable group of phenylboronic acids were explored to develop various sensing strategies, including optical, electrochemical, and other methods. However, compared to the numerous monoboronic acid molecules and methods developed for glucose monitoring, the diversity of DBA molecules and applied sensing strategies remains limited. The challenges and opportunities are also highlighted for the future of glucose sensing strategies, which need to consider practicability, advanced medical equipment fitment, patient compliance, as well as better selectivity and tolerance to interferences.

## 1. Introduction

Diabetes is a chronic disease with an inadequate ability to regulate blood glucose levels. Long-term hyperglycemia can cause severe damage to organs, such as the heart, blood vessels, eyes, kidneys, and nerves, leading to a higher risk of premature death [1,2,3,4,5]. While there is no cure for diabetes, blood glucose level management can significantly reduce the health risks [6]. Affordable home glucose monitoring, particularly with stable real-time glucose level tracking ability, is an effective way to guide the management and improve the life quality of diabetics [7]. Currently, most commercial glucose monitoring sensors are enzyme-based devices, such as glucose oxidase and glucose dehydrogenase, relying on electrochemical analytical measurements. Despite the good selectivity and sensitivity toward glucose, enzyme-based strategies have some drawbacks, especially for continuous glucose monitoring, such as inflated cost, thermal and chemical instability, and strict storage conditions [8,9]. Enzyme-based sensors require complex enzyme fixation processes to maintain enzyme activity, and the commercialized manufacture of enzyme-based devices requires sterilization of the entire production line [10]. In contrast, non-enzymatic sensors without biological units are advantaged in terms of structural simplicity, quality control for mass production, and better stability under different storage and usage conditions. Therefore, there is a strong demand for the development of non-enzymatic sensors with long-term stability and low cost.

Until now, several non-enzymatic strategies have been developed, such as catalytic nanomaterials-based non-enzymatic electrochemical sensors, metabolic heat conformation methods, and near-infrared or terahertz optical sensing methods [11,12,13]. Nanomaterials-based electrochemical sensors have been extensively studied, but most of the sensors have to work in harsh conditions (e.g., alkali solution). Other strategies are promising alternatives but are challenged by selectivity and accuracy [14]. Aromatic boronic acid (BA)-derivatives-based glucose sensing is another alternative, relying on their dynamic covalent binding with diol moieties of saccharides. The dynamic binding property offers the possibility to develop continuous glucose monitoring devices using various sensing methods. However, the lack of selectivity to glucose in BA-based glucose sensing was the biggest obstacle to practical application.

The binding affinity between boronic acid and saccharide is strongly dependent on the orientation and arrangement of hydroxyl groups in saccharide, enabling the differentiation of various monosaccharides. Glucose contains two binding sites with boronic acid, cis-1,2-dihydroxyl and cis-3,5,6-trihydroxyl groups. Therefore, the establishment of diboronic acid derivatives with two recognition sites can achieve selective recognition of glucose, and the design of suitable diboronic acid (DBA) structures has become a hot research topic in past decades.

**Figure 1:**
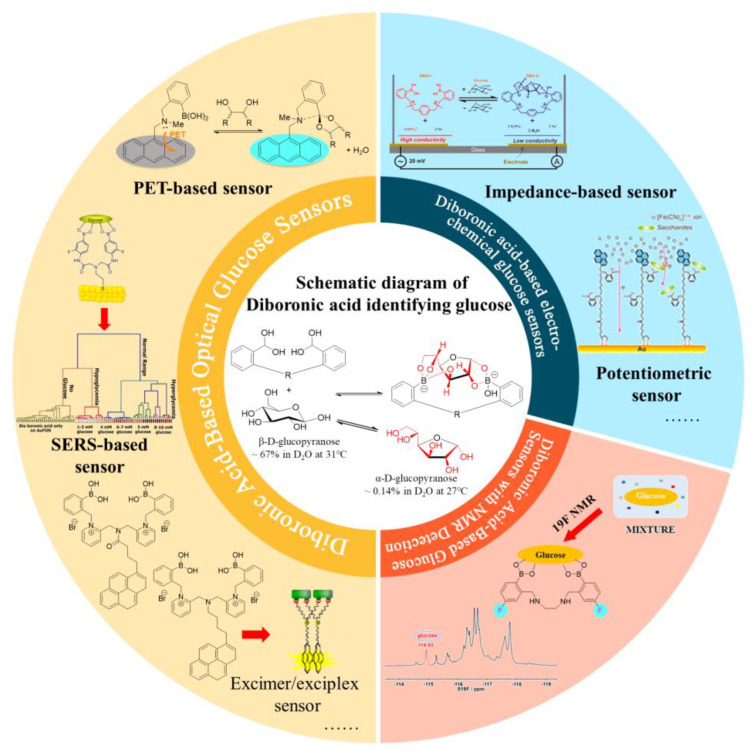
Common detection methods of diboronic-acid-based glucose sensors in recent years.

Though the development of boronic acid-based glucose monitoring has been frequently summarized, most of them cover all boronic acid sensors, and quite a few reviews have focused on DBA-based sensing strategies, which are more practical [15,16,17,18]. Herein, in this review, we emphasize the fundamental mechanisms involved in boronic acid-based glucose sensing strategies, especially focusing on DBA-based glucose sensors reported in the past 10 years (Figure 1).

## 2. Mechanism of Selective Glucose Recognition via Diboronic-Acid-Based Sensors

**Figure 2:**
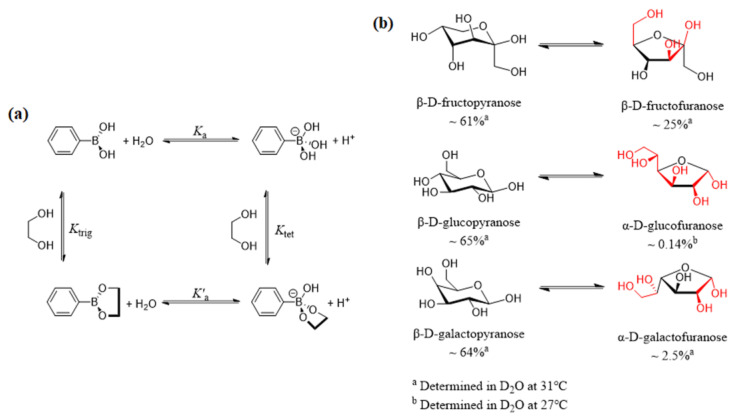
(**a**) Binding equilibria of phenylboronic acid with a diol [20]; (**b**) Equilibrium between the dominant form (pyranose, left) and the form that contains a syn-periplanar anomeric hydroxyl pair (furanose, right) of D-fructose, D-glucose, and D-galactose [21].

The reversible and covalent reaction between boronic acids and saccharides with cis-1,2- or 1,3-diol moieties has been studied in detail. In 1959, Lorand and Edwards quantified the interaction between phenylboronic acids and diols based on the change of pH in a solution, and the pH-dependent thermodynamic cycle of the reversible reaction is shown in Figure 2a [19,20]. In an aqueous solution, phenylboronic acid can react with water, showing an equilibrium process (*K*_a_) between the planar triangular structure of boronic acid and the tetrahedral boronate anion structure. Generally, the binding strength of the boronate anion (*K*_tet_) to the diol is larger than that of boronic acid (*K*_trig_), resulting in a decrease in boronic acid p*K*_a_ values by 2–3 units.

Saccharides may exist in two structural configurations, open chain and cyclic. For monosaccharides, cyclic structures contain furanose (five-membered) or pyranose (six-membered). Boronic acids tend to bind the furanose form, and a high proportion of furanose conformation contributes to a higher binding constant. As shown in Figure 2b, fructose and galactose contain ~25% and 2.5% furanose form, respectively, while glucofuranose is only ~0.14% [21]. Therefore, boronic acid traditionally shows a relatively low affinity to glucose, and other saccharides severely disturb the precision of monoboronic-acid-based glucose sensing [22].

**Figure 3:**
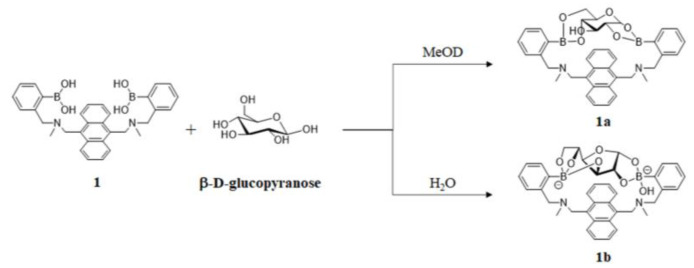
D-glucose was bound with sensor **1** in the β-pyranose form in deuterated methanol [23] and in the α-furanose form in basic aqueous media [24].

Shinkai et al. developed the first glucose-selective fluorescent diboronic acid sensor **1** in 1994 and proposed a 1:1 binding mechanism between the two boronic acid moieties and two potential binding sites of glucopyranose (Figure 3) [23]. The binding constant of sensor **1** to glucose, measured in 33.3% methanol/H_2_O at pH 7.77, was 3981 M^−1^, which was much higher than that of fructose (316 M^−1^) and galactose (158 M^−1^). Subsequently, Norrid and Eggert re-examined this work and re-confirmed the conformation of complex **1a** under anhydrous conditions. Furthermore, in the presence of water, complex **1a** rearranges into the thermodynamically more stable complex **1b**, where diboronic acid interacts with α-D-glucofuranose by binding to the cis-dihydroxyl groups at position 1,2 or cis-trihydroxyl groups at positions 3,5,6 (Figure 3) [24]. The glucose-selective diboronic acids share a similar structural arrangement in that the two boronic acid moieties match both the distance between the two potential binding sites of glucose and the orientation of the hydroxyl groups.

Based on this property, researchers have designed different structures of diboronic acids to improve the selectivity for glucose, enabling their application in glucose detection. In addition, the *pseudo*-diboronic acid moieties designed by the assembly of two monoboronic acid molecules at precise positions are also able to recognize the two 1,2-diol moieties of D-glucose, such as the supramolecular inclusion complex of γ-cyclodextrin (γ-CD) with two molecules of monoboronic-acid-based receptors [25,26,27]. However, we will not cover *pseudo*-diboronic acids in this review as we focus on sensors that possess two boronic acid moieties in one single molecule.

## 3. Design Principles of Diboronic-Acid-Based Optical Glucose Sensors

In previous decades, optical sensors have gained significant attention due to their high sensitivity. In biosensing, a variety of sensing mechanisms have been applied in diboronic-acid-based sensors, including intramolecular charge transfer (ICT), photoinduced electron transfer (PET), fluorescence resonance energy transfer (FRET), excimer/exciplex, and surface-enhanced Raman spectroscopy (SERS).

### 3.1. Intramolecular Charge Transfer (ICT) Sensors

**Figure 4:**
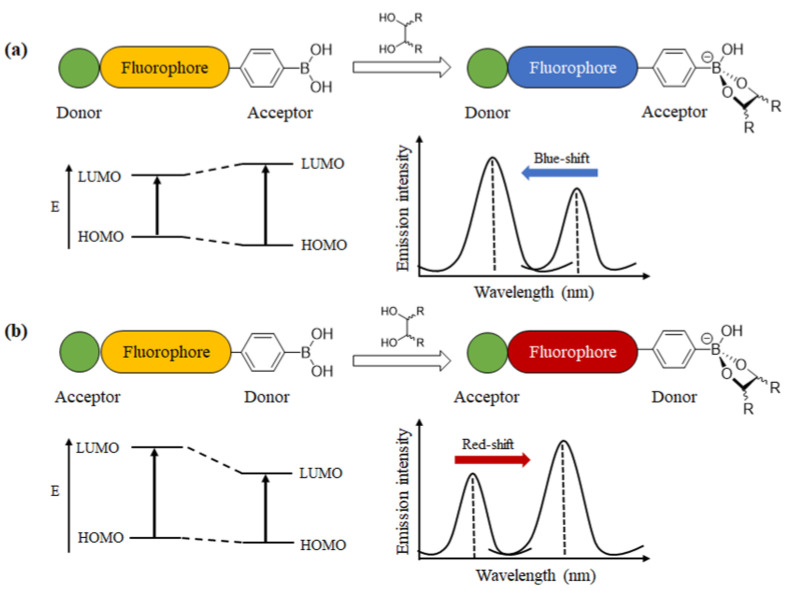
Schematic diagram of intramolecular charge transfer. The recognition of cis-diols with phenylboronic acid-conjugated ICT fluorophores may result in a blue-shift (**a**) or red-shift (**b**) of fluorescence emission depending on the structure of different molecular structures.

Intramolecular charge transfer (ICT) sensors are a class of fluorescent molecules that possess a push–pull electron system, typically comprising an electron-accepting group (A) and an electron-donating group (D). The change of electron distribution in the D-A system can lead to the shift of fluorescence emission spectra along with the variation in emission intensity (Figure 4) [28]. Fluorophores with boronic acid groups were found to respond to molecules with diol groups.

**Figure 5:**
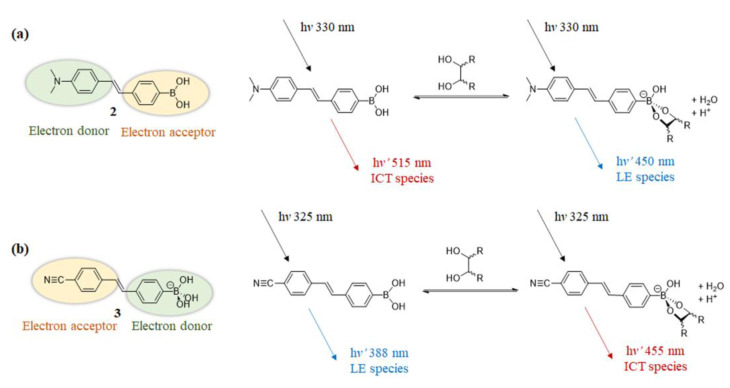
(**a**) The emission wavelength of sensor **2** blueshifts due to the interruption of the ICT state when saccharide is added (or an increase in pH); (**b**) The emission wavelength of sensor **3** redshifts due to the interruption of the ICT state when saccharide is added (or an increase in pH). (Colors are used to describe the red and blue shift in the emission wavelengths, not the actual color of the emitted light.)

To explore the fluorescence response mechanism of boronic acid-based sensors, DiCesare and Lakowicz examined a series of stilbene-4-boronic acid derivatives by introducing electron-accepting or electron-donating groups at the 4′ position [29,30]. In the case of sensor **2** with the 4′-dimethylamino as the donor group, when boron is *sp*^2^ hybridized and acts as an acceptor, excited-state ICT can occur between the amino donor and boron acceptor, redshifting the emission wavelength of *sp*^2^ species. While boron is *sp*^3^ re-hybridized, its acceptor abilities are lost. This results in a change in the ICT effect and shifts the emission wavelength of the fluorophore toward higher energies. For the *sp*^2^ to *sp*^3^ interconversion, a blue shift of 45 nm is induced in the emission wavelength, accompanied by an increase in emission intensity (Figure 5a). By increasing the pH of the solution of sensor **2** (from 6.0 to 12.0) or adding saccharides to the buffer solution at pH 8.0 to generate *sp*^3^ species, the fluorescence response was exactly the same. For comparison, they designed 4′-cyanostilbene-4-boronic acid (**3**), with the strong electron-withdrawing cyano group as the electron acceptor. When boron is *sp*^3^ re-hybridized, the boron is no longer an acceptor group, allowing the changes of ICT and red-shifted emission (Figure 5b). By examination of two diametrically opposed systems, they verified the change in the electronic properties of the sensor after the binding to saccharides occurs via the ICT effect [31].

Although ICT is an important mechanism in mono-boronic acid-based optical sensors, it has not been reported in the molecular design of diboronic-acid-based glucose sensors. This is probably because the introduction of optical moieties to boronic acid results in complicated synthesis and poor water solubility.

### 3.2. Photoinduced Electron Transfer (PET) Sensors

**Figure 6:**
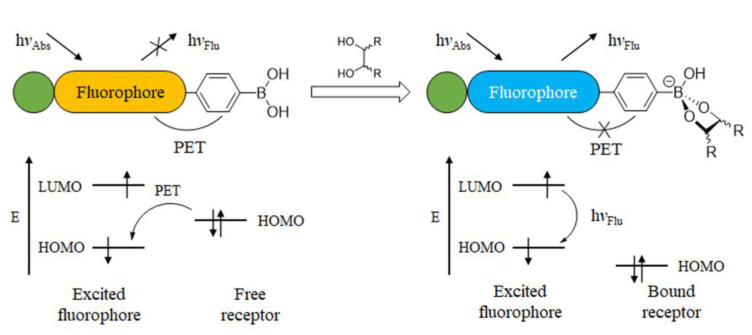
Schematic diagram of photoinduced electron transfer.

The most widely reported optical glucose sensors are designed based on the PET effect, which typically involves a fluorophore connected to the amine group of (2-(aminomethyl)phenyl)boronic acid with a methylene spacer [32]. When the fluorophore is excited, an electron from the highest occupied molecular orbital (HOMO) is promoted to the lowest unoccupied molecular orbital (LUMO), followed by electron transfer from the HOMO of the donor (free amine) to the HOMO of the fluorophore (Figure 6). This process results in fluorescence quenching (OFF). However, when the sensor binds to a saccharide molecule, the amine group interacts strongly with the boron atom, which increases the redox potential of the donor and lowers the energy of the associated HOMO. As a result, the PET process is hindered, and the fluorescence is recovered (ON). Therefore, this system functions as a molecular switch turned on by the analytes.

#### Intra-Molecular PET Sensors

**Figure 7:**
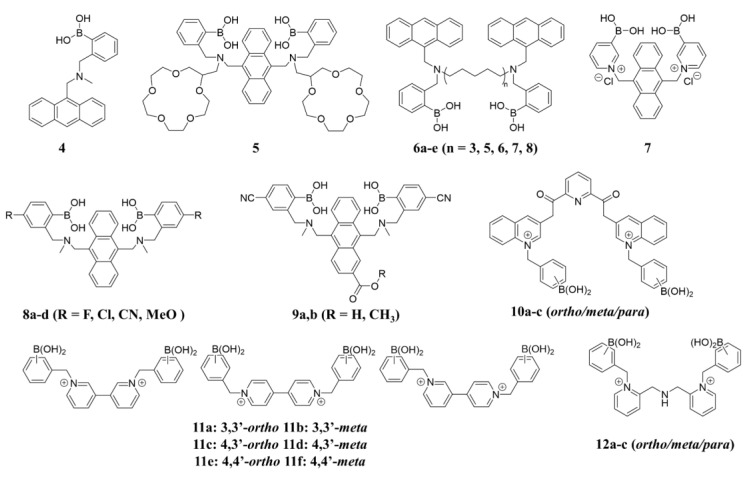
The chemical structures of PET sensors **4**–**12**.

**Figure 8:**
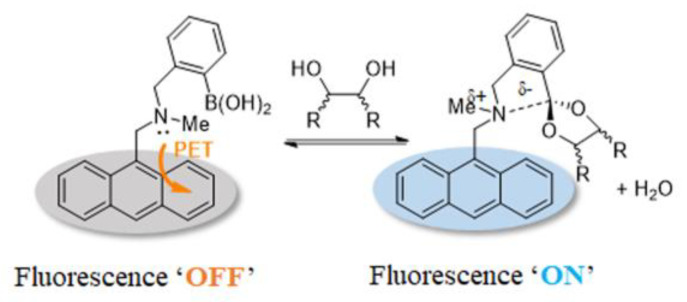
Illustration of the anthracene-based PET system.

In 1994, Shinkai reported the first PET-based fluorescent glucose sensor (sensor **4,** structure shown in Figure 7), which consisted of a benzylamine with a boronic acid group attached at the adjacent position and a fluorescent group, anthracene [33]. As shown in Figure 8, the B-N interaction between the amino nitrogen atom and the boronic acid group is very weak in the absence of the substance, and the lone electron pair on the nitrogen can quench the fluorescence of anthracene by a PET process, so the sensor is in the “off” state. However, the complexation with saccharide increases the acidity of the boronic acid group on sensor **4**, and a stronger Lewis interaction between the boron atom and the lone pair of the amine can occur. The B-N interaction triggers the recovery of the inherent fluorescence of anthracene and then is in the “on” state [34,35,36]. However, Sun and coworkers determined that the vibrational-coupled excited-state relaxation instead of B-N interaction plays an important role in the modulation of the fluorophore’s fluorescence [37].

Sensor **1** with two boronic acids was designed with a higher selectivity toward glucose than fructose by Shinkai [23]. The sensor contains two boronic acid receptors, and the spatial spacing of the diboronic acid groups provides an effective binding pocket for glucose. The binding constant of sensor **1** to glucose in a water/methanol buffer at pH = 7.8 was 3980 M^−1^, which is 12-fold greater than that of fructose (316 M^−1^) and 25-fold greater than that of galactose (158 M^−1^). James and Shinkai et al. decorated 15-crown-5 rings to sensor **1** and obtained sensor **5**, which exhibits unique “glucose cleft” and “metal sandwich” properties [38]. When Na^+^, K^+^, Sr^2+^, and Ba^2+^ are present, two 15-crown-5 rings form a metal sandwich, making sensor **5** impossible to form the 1:1 diboronic acid-glucose complex due to the increased spacing between the two boronic acids. This work indicated that the spatial disposition of the two boronic acid moieties is crucial for preferential binding to glucose. Since then, diboronic acid sensors with an anthracene ring based on the PET mechanism have been extensively studied. Lots of researchers have optimized the molecular structure of sensor **1**. Wang and coworkers designed a series of fluorescent sensors in 2017, designated as **6a**–**e** [39]. Linkers with different lengths, molecule rigidity, and boronic acid spatial orientations were introduced to optimize the arrangements of the two boronic acid units, which has improved selectivity for mono-/oligosaccharides. The fluorescence intensity of **6a**–**e** increased significantly with the addition of various monosaccharides/bisaccharides. Compounds **6d** and **6e** showed strong binding affinities but poor selectivity to glucose and fructose (**6d**: *K*_glu_ = 1418 M^−1^, *K*_fru_ = 1666 M^−1^; **6e**: *K*_glu_ = 1990 M^−1^, *K*_fru_ = 1896 M^−1^). Furthermore, the Eversense, which uses sensor **1** as a fluorescent probe for continuous glucose monitoring, is the only FDA-approved and successfully commercialized diboronic-acid-based glucose sensor that provides 180 days of continuous glucose monitoring in humans. This continuous glucose monitoring system measures glucose in the interstitial fluid every 5 min and requires daily fingertip blood collection calibration [40,41,42,43].

**Figure 9:**
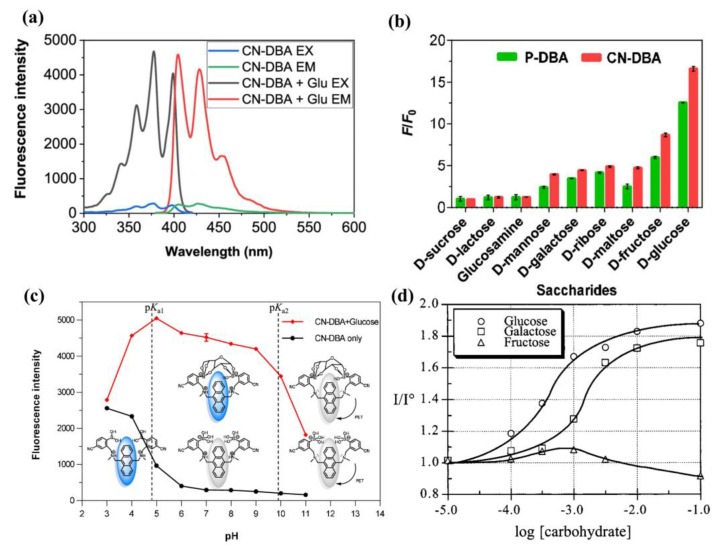
(**a**) Fluorescence excitation and emission spectra of CN-DBA (10 μM in 0.5% MeOH/PBS, pH = 7.4) before and after adding glucose (0.1 M) (*λ*_ex_ = 375 nm, *λ*_em_ = 427 nm); (**b**) Fluorescence changes (F/F_0_) of P-DBA and CN-DBA in 1.56 mM saccharides; (**c**) Illustration of the fluorescence responses of CN-DBA against 0.1 M glucose under different pH values. Reprinted with permission from [45]. Copyright © 2021 American Chemical Society; (**d**) Relative fluorescence of sensor **7** (10^−5^ M, 0.05 M aqueous phosphate buffer, pH 7.4) as a function of carbohydrate concentration [44]. Reprinted with permission from [44]. Copyright © 1999 American Chemical Society.

Although the introduction of the diboronic acid structure greatly enhances the selectivity for glucose, the sensors are mostly poorly water-soluble, which limits practical applications in aqueous media and at physiological pH. To solve the issue, Eggert and his colleagues synthesized sensor **7** based on sensor **1** by replacing the benzene ring with a positively charged pyridinium group [44]. The cationic pyridinium salt in the sensor provides a p*K*_a_ of only 4.0, which benefits the binding of the sensor to glucose in neutral aqueous solutions. Binding studies in aqueous solutions at physiological pH showed that the sensor binds to glucose (1:1) in the form of α-D-glucofuranose at positions cis-1, 2 and cis-3, 5. As shown in Figure 9d, sensor **8** exhibits a selective fluorescence response to glucose compared to fructose and galactose (binding constant of 2512 M^−1^ with D-glucose).

To further improve water solubility, Wang and coworkers modified the parent structure of sensor **1** (P-DBA) by introducing electron-withdrawing groups (F, Cl, and CN) and electron-donating groups (MeO) at the para-position of phenylboronic acid and obtained a series of diboronic-acid-based sensors **8a**–**d** [45]. The cyano-substituted compound **8e** (CN-DBA) exhibited the highest glucose binding constant (6489.5 M^−1^, 33% MeOH/PBS) and is highly soluble in an aqueous solution containing 0.5% MeOH with a lower p*K*_a_ value (4.894) than its parent molecule P-DBA (5.667). CN-DBA can accurately detect glucose in the pH range of 6.0–9.0 and has an ultra-sensitive recognition of glucose (LOD = 1.51 μM). CN-DBA can be used as an accurate and sensitive fluorescent probe for glucose detection in biological samples, as demonstrated by its ability to detect glucose in cell lysates and plasma with good recovery and precision (Figure 9a–c).

**Figure 10:**
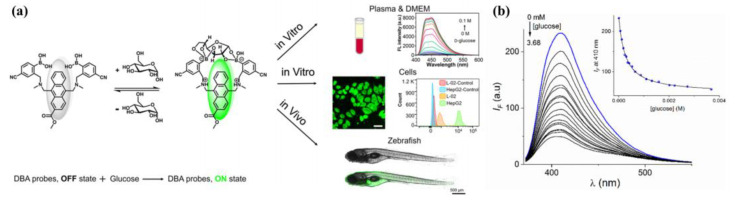
(**a**) Schematic illustration of the sensing mechanism of **9a** for glucose and its bioimaging applications in this work. Reprinted with permission from [46]. Copyright 2023 The Authors. Published by American Chemical Society; (**b**) the emission spectra of sensor **10b** with glucose (in PBS buffer/10% MeOH, pH = 7.40, *λ*_ex_ = 350 nm). Reprinted with permission from [47]. Copyright © 2023 American Chemical Society.

In a recent study, T.D. James et al. designed and synthesized two diboronic acid glucose probes Mc-CDBA(**9a**) and Ca-CDBA(**9b**) by introducing water-solubilizing group cyano (-CN) at the phenylboronic acid counterpart of the sensor **1** and methoxycarbonyl (-COOCH3) and carboxyl (-COOH) at the β-position of the anthracene ring to improve the biocompatibility (Figure 10). These two probes were successfully applied to multidimensional imaging of both cells and zebrafish, providing a technical tool for clinical medical studies of glucose homeostasis in vivo and for the study and diagnosis of metabolic diseases [46].

In addition, A.D. González et al. designed pyridine-2,6-dicarboxamide-quinoline salts **10a**–**c** containing two phenylboronic acid groups for optical recognition with good water solubility and photostability. Among them, the **10b** showed the highest affinity and selectivity for glucose (*K*_a_ = 3800 M^−1^) and good selectivity toward glucose over other monosaccharides. UV-vis and fluorescence titration experiments, HRMS measurements, X-ray crystal structure, and DFT calculations indicate that glucose binds to **10b** in a 1:1 mode via its furanose form co-bound with diboronic acid (Figure 10b). The affinity of glucose for receptor **10b** is higher than all reported cationic diboronic-acid-based related receptors in aqueous media. The p*K*_a_ values of receptor **10b** for boronic acid range from 7.7 to 7.1, dropping to ~6.2 in glucose complexation, allowing for recognition at physiological pH [47].3.2.2. Inter-Molecular PET Sensors

**Figure 11:**
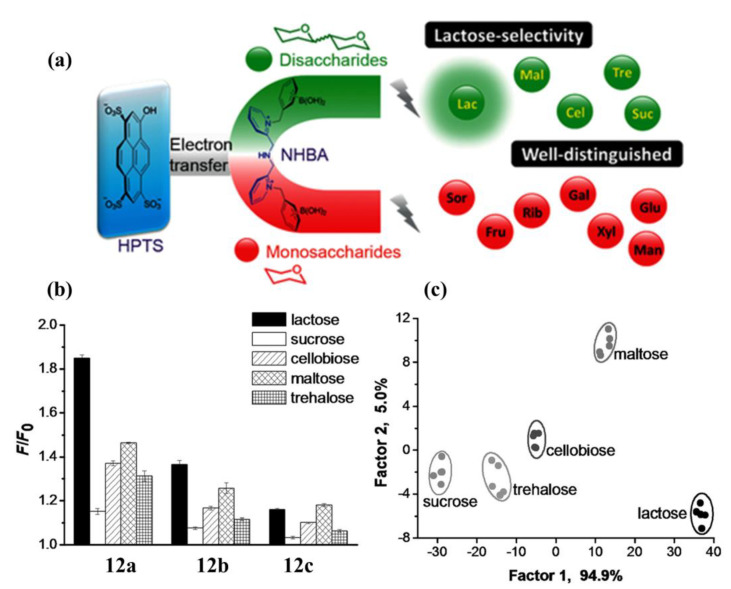
(**a**) Schematic diagram of the recognition of saccharides using inter-molecular PET system based on diboronic acid **12a**–**c** and **HPTS**; (**b**) Fluorescence response of sorbitol and different monosaccharides based on **HPTS**/**12a**–**c** ensembles at pH 7.4 using a fluorescence plate reader; (**c**) Two-dimensional canonical score plot of seven analytes (1 mM) analyzed by LDA. Reprinted with permission from [50]. Copyright © 2015 Wiley Online Library.

Different from intramolecular PET sensors, the boronic acid-based inter-molecular PET sensors consist of two components, a dye and a dye-quencher. The boronic acid molecule acts as a quencher and an acceptor for the saccharides, and the fluorescence of the dye is modulated by electron transfer from the dye to the quencher/acceptor. Singaram et al. developed an intermolecular PET sensing system using the anionic dye 8-hydroxypyrene-1,3,6-trisulfonic acid trisodium salt (**HPTS**) and cationic diboronic acid derivatives (**11**) and reported the identification of monosaccharides and disaccharides with a six-channel, two-component sensor array consisting of **11a**–**f** and **HPTS** [48,49]. In 2015, Xing and colleagues reported a series of cationic N,N-di-2-picolylamine-derived diboronic acid molecules **12a**–**c** to distinguish six monosaccharides and five disaccharides with anionic **HPTS** as the fluorescent indicator [50]. Compared with the known diboronic acid receptors based on viologen and phenanthrolinium salt, these molecules have remarkably higher spatial flexibility and a significantly larger pocket for sugar analytes. As shown in Figure 11, a well-separated two-dimensional linear discriminant analysis (LDA) plot demonstrated that the sensor array based on **12a–c** can achieve efficient discrimination of various monosaccharides.

### 3.3. Fluorescence Resonance Energy Transfer (FRET) Sensors

FRET is a non-radiative energy transfer process via a long-range dipole-dipole interaction between donor and acceptor. The process occurs when the vibrational energy difference between the ground state and the first excited state of the donor matches that of the acceptor or when the emission spectrum of the donor overlaps with the excitation spectrum of the acceptor [51]. The FRET mechanism provides an alternative approach to designing fluorescent sensors, but it has more stringent requirements in terms of the fluorescence relationship between the donor and acceptor and the spatial positioning of the boronic acid groups.

**Figure 12:**
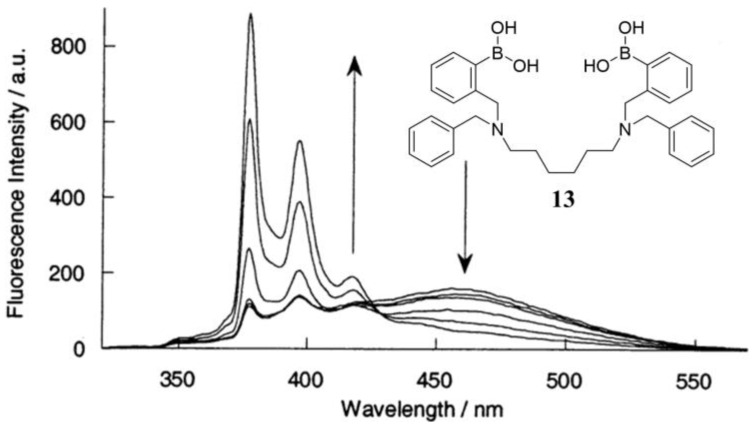
Fluorescence spectral change of **13** with different concentrations of D-glucose (2.5 μM in buffer, pH = 8.21, *λ*_ex_ = 299 nm). Reprinted with permission from [52]. Copyright © 2002 American Chemical Society.

The representative diboronic acid fluorescent sensor (**13**) using the FRET mechanism was developed by James et al. in 2002 [52]. Sensor **13** contains two phenylboronic acid groups, a hexamethylene linker, and two different fluorophore groups (phenanthrene and pyrene). The emission spectrum of phenanthrene (donor, *λ*_em_ = 369 nm) is well-overlapped with the excitation spectrum of pyrene (acceptor, *λ*_ex_ = 342 nm), which offers the basis of FRET from phenanthrene to pyrene in sensor **13.** Moreover, the intra-molecular π-π stacking between phenanthrene and pyrene in sensor **13** also contributes a long wavelength exciplex emission at 460 nm (Figure 12). Upon binding with glucose, the exciplex emission decreased, and the emission from pyrene was significantly enhanced under 299 nm excitation. This result indicates the break of the intramolecular π-π stacking and efficient FRET from phenanthrene to pyrene. The fluorescence enhancement of sensor **13** by D-glucose is 3.9 times, while the enhancement is 1.9 times by D-fructose. These results indicate that the energy transfer from phenanthrene (donor) to pyrene (acceptor) in a rigid 1:1 cyclic D-glucose complex is more efficient than in a flexible 2:1 acyclic D-fructose complex.

### 3.4. Excimer/Exciplex Sensors

An excimer/exciplex is a homodimeric or heterodimeric complex of two fluorophores that interact through π-π interaction, with one in the excited state and the other in the ground state. These complexes usually emit a red-shifted broad peak compared to the individual monomers. The substrate recognition of excimer/exciplex-based sensors relies on the changes of π-π interactions upon receptor binding, which can cause either an increase or a decrease in fluorescence intensity [53].

**Figure 13:**
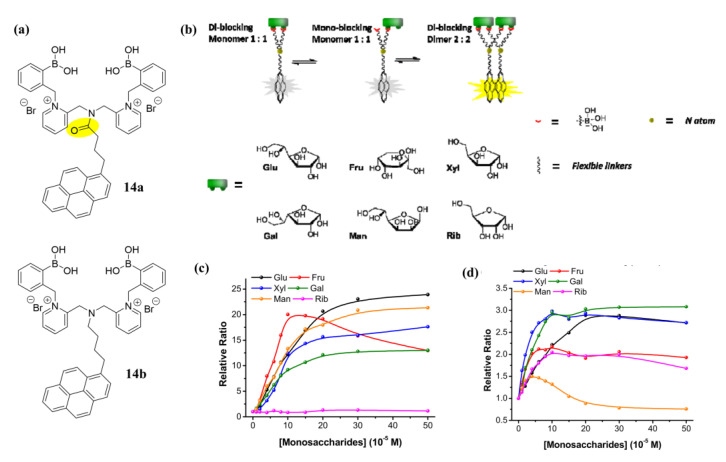
(**a**) Molecular structure of sensor **14a** and **14b**; (**b**) Hypothetical binding modes between diboronic acids and monosaccharides; (**c**,**d**) Relative ratios of fluorescence signals towards monosaccharides using **14a** (**c**) or **14b** (**d**) as a sensor at pH 10.0. Reprinted with permission from [54]. Copyright © 2016 American Chemical Society.

In 2016, Xing et al. reported the synthesis of two water-soluble excimer-based sensors **14a**–**b** that differ only in their flexible aliphatic linkers between the fluorophore pyrene and the diboronic acid moiety [54]. Both **14a** and **14b** can form pyrene excimers in the presence of monosaccharides. Monomers **14a** and **14b** had fluorescence emissions at 381 nm, while distinct excimer emissions at 528 and 510 nm, respectively (Figure 13). The two sensors showed different responses to monosaccharides. They also built a four-channel assay using linear discriminant analysis (LDA) to distinguish six monosaccharides within a certain concentration range by monitoring monomer and excimer emissions. This assay accurately detected glucose in artificial urine and blood containing common interferents, proving the effectiveness of the system under physiological conditions.

### 3.5. Surface-Enhanced Raman Spectroscopy Sensors

Surface-enhanced Raman spectroscopy (SERS) is an analytical technique that has been widely used since the 1980s for its high sensitivity and selectivity to low concentrations of analytes. The SERS enhances the Rama signal by generating a strong electric field when molecules are adsorbed to metallic surfaces with nanometric features [55]. However, the electric field decays rapidly from the substrate surface, resulting in a strong distance dependence. Therefore, the molecule to be detected must be within 2 nm of the nanostructured substrate surface to be efficiently detected [56].

**Figure 14:**
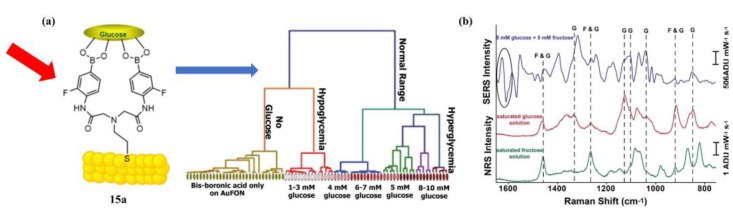
(**a**) Greater distinction between spectra can be found when coupling hierarchical cluster analysis (HCA) with PCA. The hypoglycemia (1–3 mM), normal (4–8 mM), and hyperglycemia (>8 mM) can be distinguished by the HCA; (**b**) SERS difference spectrum of a 5 mM glucose and 5 mM fructose mixture (purple), the normal Raman spectra of a saturated glucose solution (red) and fructose (green). The dashed lines indicate peaks in the SERS spectrum that arise from glucose or fructose. Reprinted with permission from [57]. Copyright © 2016 American Chemical Society.

In 2016, Sharma and coworkers developed SERS sensors **15a**–**e**, a diboronic acid-modified gold film-over-nanosphere (AuFON) substrates for direct detection of glucose [57]. A panel of diboronic acid analogues with variable length linkers was synthesized and exhibited higher glucose binding affinities than 4-amino-3-fluorophenylboronic acid (Table 1). Surprisingly, they selected the *n,n* = 1,1-diboronic acid (**15a**) for SERS studies (for **15a**, *K*_glu_/*K*_fru_ = 0.10). In the presence of fructose and glucose, the SERS spectra of the diboronic acid immobilized AuFON exhibited distinct line shapes, enabling the differentiation of various monosaccharides. Additionally, by incorporating principal component analysis (PCA) with the SERS data, the above-mentioned SERS sensor can distinguish well between hypoglycemia, hyperglycemia, and normal glucose levels (Figure 14). Therefore, the SERS-based strategy is promising for the further development of in vivo glucose sensors.

### 3.6. Vibration-Induced Emission (VIE)-Based Sensors

Vibration-induced emission (VIE) is a new mechanism proposed by Tian and coworkers when studying the dynamic luminescence properties of dihydrophenoxazine molecules. For the saddle-shaped dihydrophenoxazine molecule, when excited by light, the aromatic rings on both sides of the N-N axis will gradually evolve to quasi-planar structures along the axis, thus exhibiting a Stokes shift of up to 250 nm; at the same time, through precise chemical modification or external environmental modulation, the dihydrophenoxazine conjugated molecular backbone can achieve conformation-dependent multi-color luminescence [58,59]. Therefore, the VIE strategy has been applied in different analytical assays; for example, Sessler and Stang have developed receptors for the discrimination of carboxylic acids in organic solvents [60].

**Figure 15:**
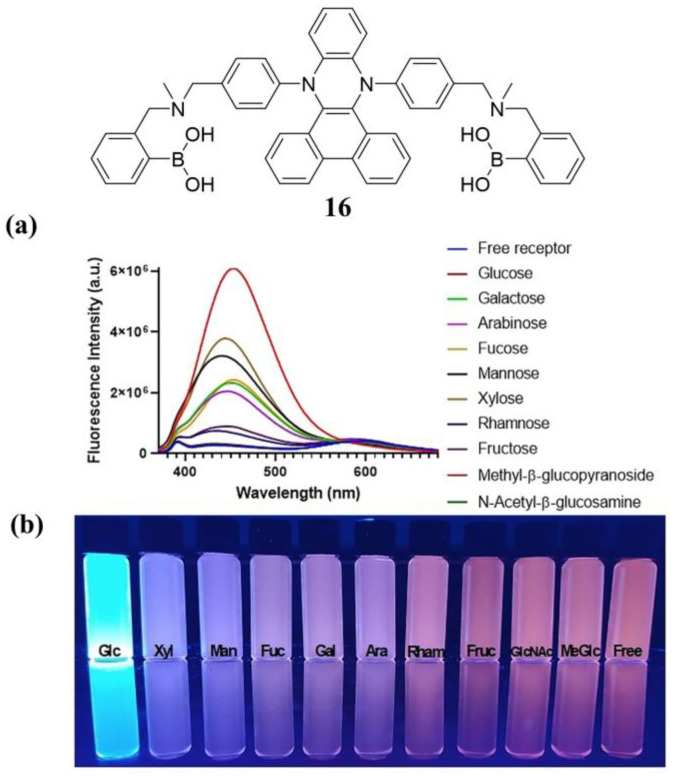
Fluorescence emission spectra (**a**) of **16** in the presence (as well as absence) of various monosaccharides at the end of titrations associated with fluorescence image under irradiation with 365 nm UV light (**b**). Reprinted with permission from [61]. Copyright © 2021 Wiley OnlineLibrary.

In 2021, Ramos-Soriano et al. reported a fluorescent diboronic-acid-based chemical sensor **16** that displays typical VIE properties. This system can distinguish monosaccharides in a mixed solvent of methanol/water (80/20), causing significant changes in fluorescence that can be distinguished by the naked eye. In the case of glucose, the recognition process leads to the most dramatic changes in color and fluorescence intensity, even in the presence of other monosaccharides, with an LOD of 9.4 μM for D-glucose (Figure 15). However, sensor **16** is insoluble in water and is less selective for D-glucose than other monosaccharides with two 1,2-diol fractions (e.g., D-galactose) [61]. This proof-of-concept study is the first example of dihydrophenoxazine-based differentiation of saccharides in aqueous media, opening new directions for the development of optical sensors for the detection of glucose using different detection principles.

## 4. Design Principles of Diboronic-Acid-Based Electrochemical Glucose Sensors

Electrochemical sensors use chemically modified electrodes as working electrodes and detect electrical signals proportional to the concentration of the substance to be measured. There are four categories of electrochemical sensors according to their output signals: potentiometric, conductometric, voltammetric, and amperometric glucose sensors. Amperometric glucose sensors are the most widely studied. They use the magnitude of the oxidation current generated by the direct catalytic oxidation of glucose on the electrode surface to determine the concentration of glucose [62,63]. Potentiometric glucose sensors detect glucose through potential changes caused by the glucose binding with recognition molecules. The reaction with glucose results in concentration differences between electrolytes on both sides of the ion-selective membrane [64]. Conductometric glucose sensors detect glucose levels by monitoring the conductivity change of the solution due to glucose reaction.

**Figure 16:**
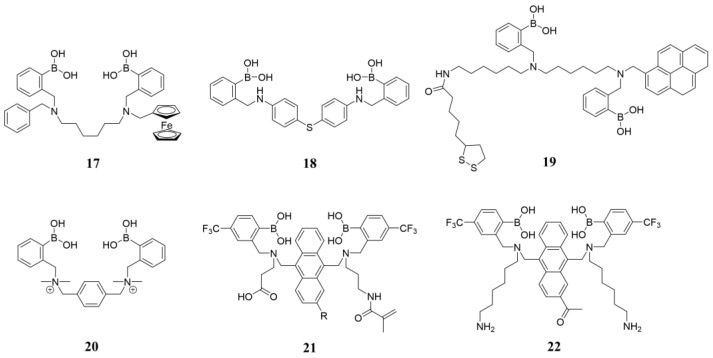
The chemical structures of electrochemical sensors **17**–**22**.

**Figure 17:**
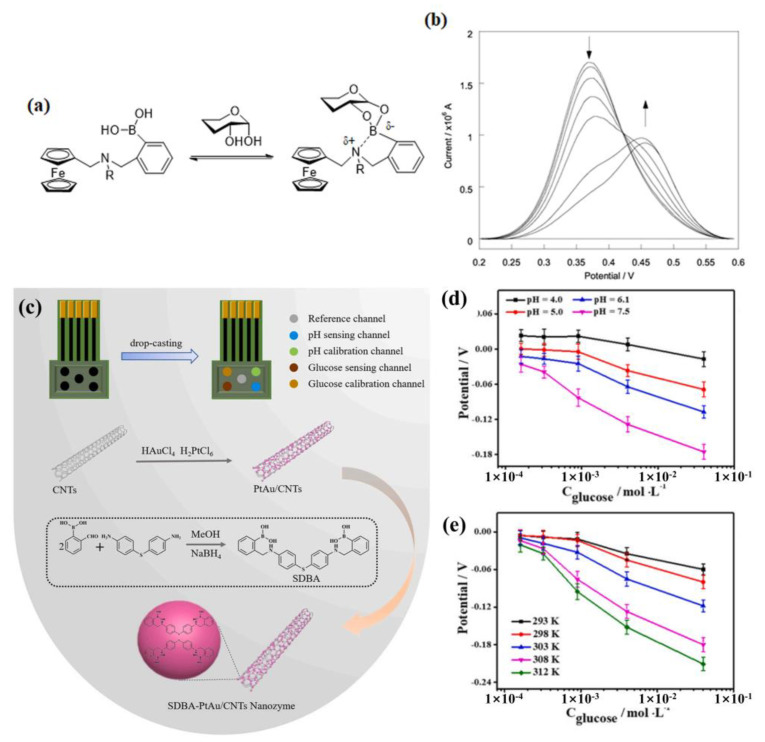
(**a**) Schematic diagram of the ferrocene-boronic acid bound to saccharide; (**b**) The DPV of **17** with different concentrations of D-glucose (50 μM in 52.1 wt.% methanol, pH 8.21). Reprinted with permission from [65]. Copyright © 2002 Royal Society of Chemistry; (**c**) Schematic illustration of MCGP sensing array based on SDBA-PtAu/CNTs nanozyme; (**d**,**e**) Response performances of glucose electrode group to glucose at different pH (**d**) and temperature (**e**). Reprinted with permission from [66]. Copyright © 2022 Elsevier B.V.

As most diboronic acids are not electrochemically active and lack redox signals within the effective potential range, it is necessary to add redox-active groups (such as ferrocene), which can be in the solution or modified on diboronic acid derivatives. Arimori and coworkers prepared a ferrocene-modified diboronic-acid-based glucose sensor **17** (structure shown in Figure 16). Differential pulse voltammograms (DPV) of **17** were recorded in the presence of different concentrations of glucose. The interaction of the boronic acid and neighboring amine is strengthened on saccharide binding, thereby reducing the electron density on the neighboring amine (Figure 17a). This, in turn, destabilizes the ferrocenium ion at higher concentrations of saccharides, resulting in a more anodic ferrocene oxidation overpotential (Figure 17b). Sensor **17** showed a 40-fold binding capacity compared to the ferrocene-modified monoboronic acid analogue to D-glucose [65].

Recently, F. Wang et al. designed a PtAu/CNTs nanoenzyme modified by diboronic acid molecules (SDBA, sensor **18**) through reversible complexation between SDBA molecules and cis-diols [66]. After SDBA modification, some of the active sites of PtAu/CNTs nanoenzymes were occupied, which reduced the catalytic activity. However, the special attraction of SDBA to glucose greatly improved the selectivity of the SDBA-PtAu/CNTs glucose sensing channel and reduced the interference of other substances, thus improving the reliability of the detection. A new multi-calibration glucose potentiometric (MCGP) sensing array, including a glucose electrode set, pH electrode set, and reference electrode channel, was designed for multiple calibrations of the basic potential due to sample matrix change (Figure 17c) and the response slope due to sample pH change (Figure 17d), thus improving the reliability of the assay. Additionally, the effect of temperature on slope has been calibrated in real time using an external temperature probe (Figure 17e). The MCGP sensing array has been further applied to the detection of glucose in human urine, and the satisfactory accuracy and reproducibility indicate that the MCGP sensing array can directly detect glucose and pH in urine without pretreatment.

**Figure 18:**
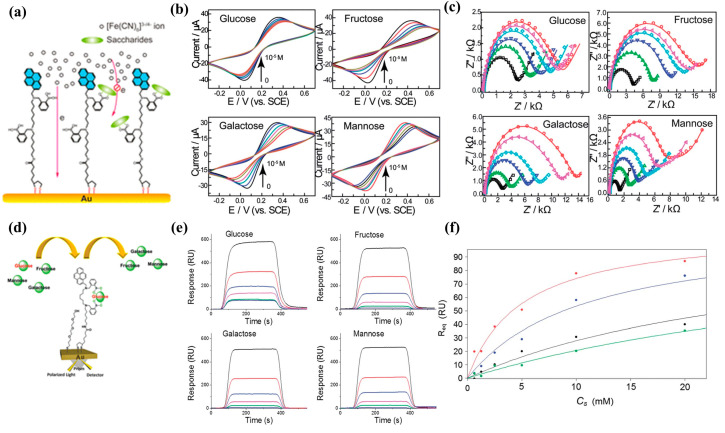
(**a**) Representation of gold surface functionalization by **19** and saccharide binding; (**b**,**c**) The detection of saccharides by CV (**b**) and EIS (**c**) (in PBS containing 5 mM Fe(CN)_6_^3−/4−^ (1:1) with 0.1 M KNO_3_, pH = 8.0). Reprinted with permission from [68]. Copyright © 2013 Royal Society of Chemistry; (**d**) Schematic diagram of SPR detection glucose; (**e**) SPR kinetic measurements detection of different saccharides concentrations (D-glucose, D-galactose, D-fructose, and D-mannose); (**f**) The calibration curve of SPR response change for diboronic acid sensor with different saccharides (glucose (red), fructose (blue), galactose (black), and mannose (green)). Reprinted with permission from [69]. Copyright © 2013 Royal Society of Chemistry.

Another approach is to modify electrodes with phenylboronic acids, and the changes in electrode surface potential to glucose are used for detection [67]. For instance, James et al. synthesized a diboronic acid derivative **19** with an electrode surface-anchored unit (1,2-dithiolane) [68]. As shown in Figure 18a–c, the diboronic acid molecules were self-assembled on the surface of a gold electrode. The anodic current of cyclic voltammetry (CV) and the charge-transfer resistance (R_ct_) of electrochemical impedance spectroscopy (EIS) was linearly related to the concentration of monosaccharides in the range of 0–10 mM in PBS and was used to evaluate the binding capability of the sensor to glucose, fructose, galactose, and mannose. The sensor exhibited good selectivity to glucose (1.7 ± 0.3 × 10^5^ M^−1^) over other monosaccharides (D-galactose 9.1 ± 1.2 × 10^4^ M^−1^, D-fructose 4.6 ± 0.5 × 10^4^ M^−1^ and D-mannose 1.2 ± 0.1 × 10^2^ M^−1^). At the same time, their partner paper uses the surface plasmon resonance (SPR) detection regime to probe the saccharide binding (Figure 18d–f) and is shown to detect D-glucose with high selectivity, showing higher affinity than the other sugars detected (i.e., D-galactose, D-fructose, and D-mannose) [69].

**Figure 19:**
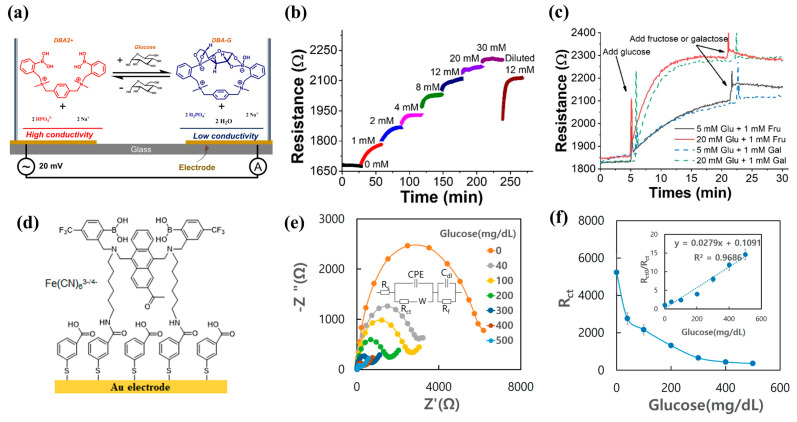
(**a**) Schematic device architecture for impedance spectra and time resolution monitoring at high frequency; (**b**) Solution resistance (R) of 1 mL of test solution changes with continuous addition of 0.5 M or 2 M glucose concentration (2 mM in 2.5 mM Na_3_PO_4_, pH = 7.6); (**c**) Addition of galactose had a negligible effect on solution resistance. The addition of fructose caused a 3% increase in resistance under low glucose (5 mM) conditions and only a transient increase under high glucose (20 mM) conditions. Reprinted with permission from [70]. Copyright © 2019 Wiley OnlineLibrary; (**d**) Schematic diagram of diboronic-acid-immobilized electrodes; (**e**) Nyquist plots and (**f**) electron transfer resistances obtained applying 50 µL of 0.1 × PBS (pH 7.4) containing 10 mM Fe(CN)_6_^3−/4−^, 0.1 M KCl, and glucose in the range of 0–500 mg/dL to the diboronic-acid-modified electrode. Reprinted with permission from [72]. Copyright © 2023 MDPI.

Bazan et al. designed a cationic sensor **20** to determine glucose concentration at physiological pH by monitoring solution conductivity changes [70]. Upon binding to glucose, the p*K*_a_ of sensor **20** decreased from 9.4 to 6.3, leading to deprotonation of boronic acid at physiological pH (7.4) and conductivity decrease in the solution by turning high conducive HPO_4_^2−^ to low conductive H_2_PO_4_^−^ (Figure 19a–c). They also tested the stability of the sensor to detect physiological (5 mM) and pathophysiological (20 mM) concentrations of glucose in the presence of interfering compounds such as fructose, galactose, lactose, and maltose and showed neglectable interference.

Joong-Hyun Kim et al. synthesized a trifluoromethyl-substituted diboronic-acid acetyl anthracene **21a**. The addition of an electron-withdrawing trifluoromethyl group at the para-position of phenylboronic acid (**21b**) increased the resistance to oxidation by reactive oxygen species (ROS), and the introduction of acetyl group in anthracene resulted in a larger Stokes shift up to 90 nm compared to the commercially used analogue**.** The introduced acetyl group did not affect the resistance to ROS, and the association constant for glucose (*K*_glu_ = 730 ± 18 M^−1^) is at least 3.6-fold higher than those of the interfering saccharides (*K*_mannitol_ = 213 ± 4.5 M^−1^; *K*_fructose_ = 164 ± 3.1 M^−1^; *K*_galactose_ = 55 ± 6.1 M^−1^) [71]. Subsequently, they synthesized diboronic acid derivative **22** for surface immobilization on a screen-printed gold electrode (SPGE) and performed the CV and EIS for detecting glucose in the range of 0–500 mg/dL by using the redox pair of 1:1 Fe(CN)_6_^3−/4−^ (Figure 19d–e). The CV and EIS results showed that the linear detection range of glucose was 40 to 500 mg/dL with limits of detection of 31.2 mg/dL and 21.5 mg/dL, respectively. In addition, they designed a glucose detection method without adding redox pairs to the sample by attaching an asymmetric membrane to the electrode, where the redox pairs are preloaded and dried. Since the membrane is attached to the electrode surface, allowing sample droplets to penetrate the electrode, the attached membrane can cover the electrode surface with a smaller volume of sample. The asymmetric membrane achieved 90% recovery of the electrochemical signal without adding redox pairs to the test sample and reduced the sample volume by a factor of 2.5. Since the sample volume is determined by the electrode size, the volume required to detect glucose can be easily reduced by fabricating an electrode of similar size to the commercially available glucose sensor, which is the key to the sensor prepared for clinical applications [72].

## 5. Diboronic-Acid-Based NMR Sensors

Nuclear magnetic resonance (NMR) spectroscopy is an atomic-level technique that provides information on molecular structure and spatial interactions [73]. The 19F atom is characterized by a high spin quantum number, high sensitivity, extensive chemical shift, and negligible human background signals; therefore, accurate and interference-free glucose testing in complicated biological samples can be realized using 19F-NMR. Although glucose sensors based on optics and electrochemistry can also achieve continuous blood glucose detection, they can only be used in vitro or above the dermis layer of the skin. Fluorinated sensors, however, hold the potential for whole-body glucose monitoring in vivo, relying on the low abundance of the element F and advantages of the magnetic resonance molecular imaging technique, such as noninvasiveness, quantitative results, and good imaging depth [74].

**Figure 20:**
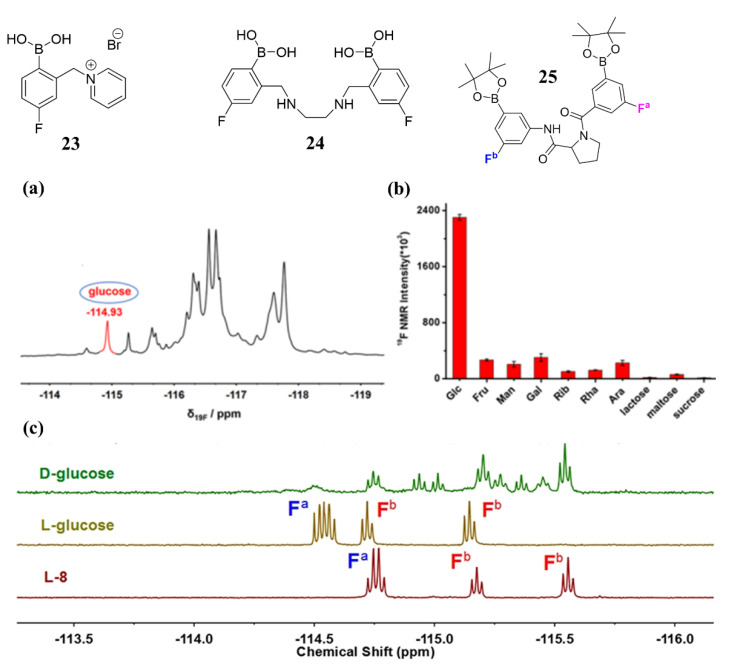
(**a**) ^19^F NMR spectrum of sensor **24** (10 mM, EtOH containing 10% D_2_O and 2 mM NaOH) in the presence of a mixture containing 10 kinds of different saccharides (10 mM for each saccharide, pH 7.4); (**b**) ^19^F NMR intensity at δ_19F_ −114.93 ppm after 10 kinds of different saccharides (10 mM for each saccharide) addition into sensor **24** (10 mM, EtOH containing 10% D_2_O and 2 mM NaOH) for 2 h, respectively. Reprinted with permission from [76]. Copyright © 2021 American Chemical Society; (**c**) ^19^F NMR spectra of sensor **25** in the presence of a D-/L-glucose in pH 9.0 buffer in D_2_O. Reprinted with permission from [77]. Copyright © 2018 American Chemical Society.

In 2017, Schiller et al. designed a water-soluble sensor **23** and realized glucose detection in synthetic urine samples merely using a low-resolution (188 MHz) NMR spectrometer with a detection limit of 1 mM [75]. However, this system only distinguishes between D-fructose and D-glucose in a binary mixture, but real human urine is a more complex system containing multiple sugars compared to synthetic urine samples. To improve the sensitivity to glucose, Shi et al. designed a fluorinated diboronic acid sensor **24** in 2021. This sensor showed a specific ^19^F NMR signal at δ_19F_ −114.93 ppm when bound to glucose, which is different from that of other saccharides [76]. Sensor **24** has shown high sensitivity and strong interference resistance for the detection of glucose, even in mixtures containing up to 10 different saccharides, and in real human urine samples without any prior treatment (Figure 20). The detection limit of this sensor for glucose in human urine samples was found to be 0.41 mM. Since the glucose concentration in the urine of a healthy person is in the range of 0~0.8 mM, ^19^F NMR-based glucose sensor **24** had the potential for the direct diagnosis of diabetes.

In addition, T.D. James et al. also reported a chiral proline-based diboronic acid by conjugating two phenylboronic acid groups at the N and C termini via amide bonds and introducing fluorine atoms on the benzene rings (sensor **25**) for chiral recognition of D-/L-glucose [77]. As shown in the ^19^F NMR spectra of sensor **25** after the addition of an excessive amount of D-/L-monosaccharides (Figure 20c), a well-resolved ^19^F NMR pattern appeared only in the presence of L-glucose resulting from the limited conformational flexibility of sensor **25**.

## 6. Conclusions and Perspective

Here, we provide a brief overview of the glucose recognition mechanism of boronic acids and discuss various glucose detection methods based on diboronic-acid sensors developed over the past decade, including optical, electrochemical, and nuclear magnetic approaches. The reversible and covalent binding between boronic acids and diols forms the basis for detecting saccharides, particularly glucose, at millimolar or sub-millimolar levels. By utilizing the specific two binding sites of glucose to boronic acid, selective glucose monitoring can be achieved using molecules containing two boronic acid groups that match both the distance and orientation of glucose’s two binding sites. However, most current diboronic acid sensors are developed through trial-and-error synthesis and screening, resulting in uneven selectivity to glucose. Despite successful cases of continuous glucose monitoring using diboronic-acid-based sensors such as Eversense being approved by the FDA, practical application in human environments remains challenging due to poor water solubility and interference from structural analogues in biological samples. Therefore, designing diboronic acid molecules with good water solubility and high glucose selectivity remains a daunting challenge for researchers.

The tunable p*K_a_*, electron-withdrawing properties, and modifiable group of phenylboronic acids have been widely used to develop various sensing strategies, including optical, electrochemical, pH-based, and other methods. Compared to the numerous monoboronic acid molecules and methods developed for glucose monitoring, the diversity of diboronic acid molecules and applied sensing strategies remains limited. Leveraging the progress made with MBA-based sensors could accelerate the development of more effective DBA sensors, as most BA and DBA sensors share similar design principles. Given the rapid development of commercial electrochemical enzyme-based glucose detection devices, combining diboronic acid with electrochemistry is a promising research direction for glucose sensors. Additionally, the development of new sensing methods, such as nuclear magnetic resonance sensing, is crucial in light of advancements in medical equipment. This may enable non-invasive whole-body glucose monitoring using fluorinated DBA [73,74,75].

Moreover, most diboronic-acid-based sensors have been investigated in non-physiological or in vitro simulated environments, with very few successfully detecting glucose in complex samples such as urine or blood. Due to the complexity of the physiological environment in the human body and the presence of interfering factors, glucose detection in actual samples or in vivo is necessary.

## Figures and Tables

**Table 1:** **15a**–**e** and monoboronic acid selectivity for glucose and fructose.

Sensors	Structures	*K*_glu_ (M^−1^)	*K*_fru_ (M^−1^)	*K*_glu_/*K*_fru_
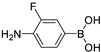	4-amino-3-fluorophenyl-boronic acid	10	200	0.05
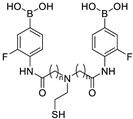	**15a**: *n,n* = 1,1	48	465	0.10
	**15b**: *n,n* = 1,2	114	389	0.29
	**15c**: *n,n* = 2,2	167	447	0.37
	**15d**: *n,n* = 2,3	99	1047	0.095
	**15e**: *n,n* = 3,3	29	379	0.077

## Data Availability

Not applicable.
